# Supplementary material for: HIV efficiently infects T cells from the endometrium and remodels them to promote systemic viral spread
Source: eLife. 2020 May 26;9:e55487. doi: 10.7554/eLife.55487 (PMC7250576; doi:10.7554/eLife.55487)
Supplement: Supplementary file 1. — Table of patient clinical parameters (age, race, cycle day, diagnosis, cycle phase, progesterone levels, and specimen source) for each of the specimens analyzed by CyTOF. [file elife-55487-supp1.docx]

**Table supplement 1. Clinical parameters of donor specimens analyzed by CyTOF**

| Donor ID | Age (years) | Race | Cycle Day | Diagnosis | Cycle Phase | Progesterone (ng/ml) | Source |
| --- | --- | --- | --- | --- | --- | --- | --- |
| 1 | 22 | AA | 36 | PCOS | Secretory | 5.12 | NMCP |
| 2 | 30 | C | 10 | PCOS | Proliferative | N/A | NMCP |
| 3 | 33 | C | 25 | No uterine pathology | Secretory | 6.82 | NMCP |
| 4 | 25 | C | 83 | PCOS | Secretory | 3.89 | NMCP |
| 5 | 36 | C | 24 | Irregular  cycles | Interval  endometrium | N/A | NMCP |
| 6 | 47 | AA | 15 | Irregular  cycles | Prolierative | N/A | NMCP |
| 7 | 39 | C | 321 | Hirsutism, Amenorrhea | Secretory | 8.29 | NMCP |
| 8 | 50 | N/A | 15 | Endometriosis, Adenomysis, Leiomyoma | Secretory | 5.4366 | UCSF |
| 9 | 37 | C | 17 | Endometriosis, Adenomysis | Secretory | 6.9495 | UCSF |
| 10 | 29 | C | 15 | Endometriosis, Leiomyoma | N/A | 6.56 | UCSF |
| 11 | 28 | C | N/A | Infertility, Irregular cycles, PCOS | Proliferative | N/A | NMCP |
| 12 | 34 | C | 5 | Infertility, Irregular cycles | Proliferative | 1.31 | NMCP |
| 13 | 31 | H | 1 | Irregular cycles | Proliferative | 2.36 | NMCP |

N/A: Not available
