# Supplementary material for: HIV efficiently infects T cells from the endometrium and remodels them to promote systemic viral spread
Source: eLife. 2020 May 26;9:e55487. doi: 10.7554/eLife.55487 (PMC7250576; doi:10.7554/eLife.55487)
Supplement: Supplementary file 2. — Table showing the antigen, metal label, antibody clone, and source of the CytOF antibodies used in this study. [file elife-55487-supp2.docx]

**Table supplement 2. List of CyTOF staining antibodies**

| **Antigen** | **Metal label** | **Clone** | **Vendor** |
| --- | --- | --- | --- |
| CD49d (α4) | 141Pr | 9F10 | Fluidigm |
| CD19 | 142Nd | HIB19 | Fluidigm |
| CCR5 | 144Nd | NP6G4 | Fluidigm |
| CD8 | 146Nd | RPAT8 | Fluidigm |
| CD7 | 147Sm | CD76B7 | Fluidigm |
| ICOS | 148Nd | C398.4A | Fluidigm |
| HSA | 150Nd | M1/69 | In-house |
| CD103 | 151Eu | Ber-ACT8 | Fluidigm |
| CD62L | 153Eu | DREG56 | Fluidigm |
| TIGIT | 154Sm | MBSA43 | Fluidigm |
| CCR6 | 155Gd | G034E3 | In-house |
| CD29 (β1) | 156Gd | TS2/16 | Fluidigm |
| OX40 | 158Gd | ACT35 | Fluidigm |
| CCR7 | 159Tb | G043H7 | Fluidigm |
| CD28 | 160Gd | CD28.2 | Fluidigm |
| CD45RO | 161Dy | UCHL1 | In-house |
| CD69 | 162Dy | FN50 | Fluidigm |
| CRTH2 | 163Dy | BM16 | Fluidigm |
| PD1 | 164Dy | EH12.1 | In-house |
| CD127 | 165Ho | A019D5 | Fluidigm |
| CXCR5 | 166Er | RF8B2 | In-house |
| CD27 | 167Er | L128 | Fluidigm |
| CD30 | 168Er | BerH8 | In-house |
| CD45RA | 169Tm | HI100 | Fluidigm |
| CD3 | 170Er | UCHT1 | Fluidigm |
| CD57 | 171Yb | HCD57 | In-house |
| CD38 | 172Yb | HIT2 | Fluidigm |
| α4β7 | 173Yb | Act1 | In-house |
| CD4 | 174Yb | SK3 | Fluidigm |
| CXCR4 | 175Lu | 12G5 | Fluidigm |
| CD25 | 176Yb | M-A251 | In-house |
| HLADR | 112Cd | Tu36 | Invitrogen |
| RORγt^#^ | 115Di | AFKJS-9 | In-house |
| Cleaved Caspase 3*^,#^ | 142Nd | D3E9 | Fluidigm |
| NFAT^#^ | 143Nd | D43B1 | Fluidigm |
| BIRC5^#^ | 145Nd | 91630 | In-house |
| Tbet^#^ | 149Sm | eBio4B10 (4B10) | In-house |
| Blimp1^#^ | 152Sm | 6D3 | In-house |
| CTLA4^#^ | 157Gd | 14D3 | In-house |

******Only for YM155 experiments, ^#^Intracellular antibodies*
