# Supplementary material for: HIV efficiently infects T cells from the endometrium and remodels them to promote systemic viral spread
Source: eLife. 2020 May 26;9:e55487. doi: 10.7554/eLife.55487 (PMC7250576; doi:10.7554/eLife.55487)
Supplement: Supplementary file 3. — SLIDE scores (Sen et al., 2015) for each individual specimen were reported as the mean of the remodeling ratios corresponding to each infected cell. Scores > 1.2 revealed viral-induced remodeling at 20% or more fold change. P-values correspond to the significance of the remodeling score and are calculated as described in the Methods. [file elife-55487-supp3.docx]

**Table supplement 3. SLIDE scores for individual specimens**

| Donor ID | Cell type | Number of infected cells | | Mean Ratio | P  Value |
| --- | --- | --- | --- | --- | --- |
| 1 | PBMC | 744 | 1.36988323 | | 4.35E-66 |
| 2 | PBMC | 25 | 1.34901841 | | 0.00045595 |
| 3 | PBMC | 137 | 1.38482881 | | 2.38E-13 |
| 4 | PBMC | 265 | 1.35748695 | | 7.87E-21 |
| 1 | ETs | 1215 | 1.39322405 | | 1.89E-120 |
| 2 | ETs | 246 | 1.30712183 | | 2.04E-10 |
| 3 | ETs | 1848 | 1.35497468 | | 5.42E-125 |
| 4 | ETs | 350 | 1.26657536 | | 8.75E-08 |
